# Supplementary material for: Molecular and Phylogenetic Analyses of Lumpy Skin Disease Virus (LSDV) Outbreak (2021/22) in Pakistan Indicate Involvement of a Clade 1.2 LSDV Strain
Source: Viruses. 2025 Nov 26;17(12):1546. doi: 10.3390/v17121546 (PMC12737554; doi:10.3390/v17121546)
Supplement: Supplementary file 1 [file viruses-17-01546-s001.zip › viruses-3929741-supplementary.pdf]

Supplemental Table S1. Parameters for the phylogenetic analysis in Mega11; *: Tamura (1992); **: Hasegawa et al (1985); ***: Tajima F. and Nei M. (1984).

region	Neighbor-Joining method			Maximum Likelihood method		
Frag 1	Tamura 3-parameter method *	gamma distribution (shape parameter = 0.05)	pairwise deletion option	Tamura 3-parameter method *		partial deletion option
Frag 2	Tamura 3-parameter method *	gamma distribution (shape parameter = 0.1)	pairwise deletion option	Tamura 3-parameter method *		Use all sites
Frag 3	Tamura 3-parameter method *		pairwise deletion option	Tamura 3-parameter method *		partial deletion option
Frag 4	Tamura 3-parameter method *	gamma distribution (shape parameter = 0.05)	pairwise deletion option	Tamura 3-parameter method *		partial deletion option
Frag 5	Tamura 3-parameter method *	gamma distribution (shape parameter = 0.22)	pairwise deletion option	Tamura 3-parameter method *		partial deletion option
Goff	Tamura 3-parameter method *	gamma distribution (shape parameter = 0.17)	pairwise deletion option	Hasegawa-Kishino-Yano model **	Gamma distribution (0.1)	partial deletion option
B22R	Tajima-Nei method ***	gamma distribution (shape parameter = 0.16)	pairwise deletion option	Hasegawa-Kishino-Yano model **	Gamma distribution (0.1)	partial deletion option
EEV	Tamura 3-parameter method *	gamma distribution (shape parameter = 0.05)	pairwise deletion option	Tamura 3-parameter method *	Gamma distribution (0.05)	Use all sites
P32	Tamura 3-parameter method *		pairwise deletion option	Tamura 3-parameter method *		partial deletion option
RPO30	Tajima-Nei method ***	gamma distribution (shape parameter = 0.06)	pairwise deletion option	Hasegawa-Kishino-Yano model **	Gamma distribution (0.6)	partial deletion option

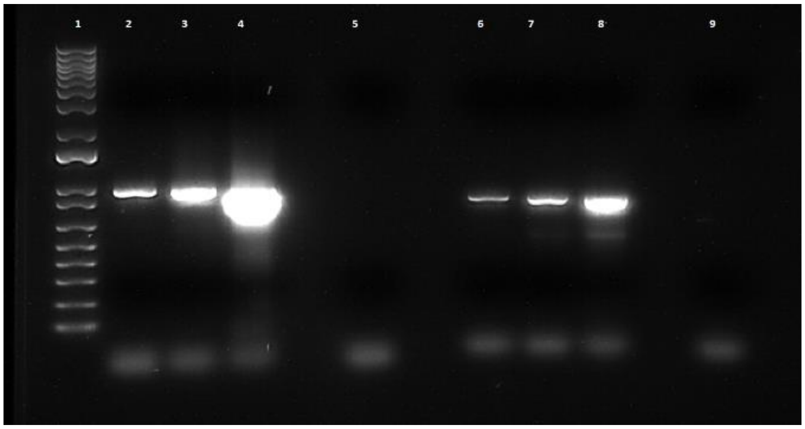

Supplementary Figure S1. Gel electrophoresis of amplified regions of selected samples; lane 1: size marker; 2 – 5: *EEV* region; 6-9: *B22R* region; 2+6: sample 1.4; 3+7: sample 3.29; 4+8: positive control; 5 + 9: negative PCR control

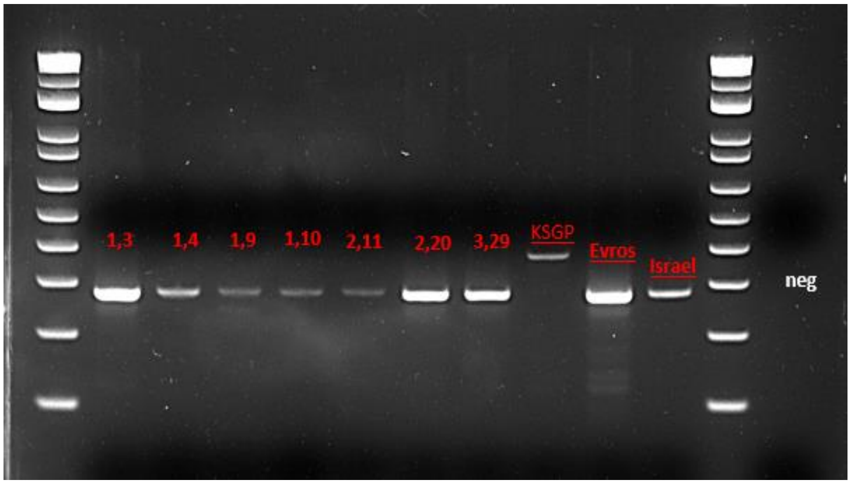

Supplementary Figure S2. Gel electrophoresis (1.5%) of the obtained amplicons. Lanes 1 and 12: Length marker (1 Kb plus); Lanes 2 to 4: Samples from Pakistan, Punjab region (Sample ID: 1.3, 1.4, 1.9); Lane 5 and 6: Samples from Pakistan, AJ&K region (Sample ID: 1.10; 2.11); Lanes 7 and 8: Samples from Pakistan, KPK region (Sample ID: 2.20 and 3.29); Lane 9: KSGP0240; Lane 10: Evros/GR/15; Lane 11: 155920/2012

```
                                              10         20         30          40          50         60

PV492548 LSDV_1_NS_2022_PAK          MADIPLYVIP IVGREISDVV PELKSDNDIF YKKVDTVKDF KNSDVNFFFK DKKDISLSYK
OK422494 LSDV/Cattle/India/2019/Ranchi MADIPLYVIP IVGREISDVV PELKSDNDIF YKKVDTVKDF KNSDVNFFFK DKKDISLSYK
KY702007 SERBIA/Bujanovac/2016        MADIPLYVIP IVGREISDVV PELKSDNDIF YKKVDTVKDF KNSDVNFFFK DKKDISLSYK
OR134834 LSDV/Albania/1707/2016       MADIPLYVIP IVGREISDVV PELKSDNDIF YKKVDTVKDF KNSDVNFFFK DKKDISLSYK
MT134042 LSDV/Russia/Udmurtiya/2019   MADIPLYVIP IVGREISDVV PELKSDNDIF YKKVDTVKDF KNSDVNFFFK DKKDISLSYK
                                     ********** ********** ********** ********** ********** **********

                                              70         80         90        100         110        120

PV492548 LSDV_1_NS_2022_PAK          FLIWEKVEKS GGVENFTEYF SGLCNALCTK EAKSSIAKHF SLWKSYADAD IKNSENKFIV
OK422494 LSDV/Cattle/India/2019/Ranchi FLIWEKVEKS GGVENFTEYF SGLCNALCTK EAKSSIAKHF SLWKSYADAD IKNSENKFIV
KY702007 SERBIA/Bujanovac/2016        FLIWEKVEKS GGVENFTEYF SGLCNALCTK EAKSSIAKHF SLWKSYADAD IKNSENKFIV
OR134834 LSDV/Albania/1707/2016       FLIWEKVEKS GGVENFTEYF SGLCNALCTK EAKSSIAKHF SLWKSYADAD IKNSENKFIV
MT134042 LSDV/Russia/Udmurtiya/2019   FLIWEKVEKS GGVENFTEYF SGLCNALCTK EAKSSIAKHF SLWKSYADAD IKNSENKFIV
                                     ********** ********** ********** ********** ********** **********

                                              130        140        150         160        170        180

PV492548  LSDV_1_NS_2022_PAK          VIEDDNTLKD LITIHNIIIEM QEKNIDIFQ LRETFHNSNS RILFNQENNNF MYSYTGGYD
OK422494  LSDV/Cattle/India/2019/Ranchi VIEDDNTLKD LITIHNIIIEM QEKNIDIFQL RETFHNSNSR ILFNQENNNF MYSYTGGYD
KY702007  SERBIA/Bujanovac/2016        VIEDDNTLKD LITIHNIIIEM QEKNIDIFQL RETFHNSNSR ILFNQENNNF MYSYTGGYD
OR134834  LSDV/Albania/1707/2016       VIEDDNTLKD LITIHNIIIEM QEKNIDIFQL RETFHNSNSR ILFNQENNNF MYSYTGGYD
MT134042  LSDV/Russia/Udmurtiya/2019   VIEDDNTLKD LITIHNIIIEM QEKNIDIFQL RETFHNSNSR ILFNQENNNF MYSYTGGYD
                                      ********** *********** ********** ********** ********** *********

                                              190        200        210        220        230        240

PV492548 LSDV_1_NS_2022_PAK          FTLSAYVIRL SSAIKIINEI IKNKGISTSL SFEMYKLEKE LKLNRQVLND SSKYILHNTK
OK422494 LSDV/Cattle/India/2019/Ranchi FTLSAYVIRL SSAIKIINEI IKNKGISTSL SFEMYKLEKE LKLNRQVLND SSKYILHNTK
KY702007 SERBIA/Bujanovac/2016        FTLSAYVIRL SSAIKIINEI IKNKGISTSL SFEMYKLEKE LKLNRQVLND SSKYILHNTK
OR134834 LSDV/Albania/1707/2016       FTLSAYVIRL SSAIKIINEI IKNKGISTSL SFEMYKLEKE LKLNRQVLND SSKYILHNTK
MT134042 LSDV/Russia/Udmurtiya/2019   FTLSAYVIRL SSAIKIINEI IKNKGISTSL SFEMYKLEKE LKLNRQVLND SSKYILHNTK
                                     ********** ********** ********** ********** ********** **********

                                              250        260        270        280        290        300

PV492548 LSDV_1_NS_2022_PAK          YLSKKRANEM KNGIWNRVGK WMAHRFPDFS YYVSHPLVSF FGIFDISIIG ALIILFIIIM
OK422494 LSDV/Cattle/India/2019/Ranchi YLSKKRANEM KNGIWNRVGK WMAHRFPDFS YYVSHPLVSF FGIFDISIIG ALIILFIIIM
KY702007 SERBIA/Bujanovac/2016        YLSKKRANEM KNGIWNRVGK WMAHRFPDFS YYVSHPLVSF FGIFDISIIG ALIILFIIIM
OR134834 LSDV/Albania/1707/2016       YLSKKRANEM KNGIWNRVGK WMAHRFPDFS YYVSHPLVSF FGIFDISIIG ALIILFIIIM
MT134042 LSDV/Russia/Udmurtiya/2019   YLSKKRANEM KNGIWNRVGK WMAHRFPDFS YYISHPLVSF FGIFDISIIG ALIILFIIIM
                                     ********** ********** ********** **:******* ********** **********
```

Supplementary Figure S3A; Amino acid sequence alignment of *P32* gene with its close relatives in phylogeny. The amino acid changes are highlighted in yellow color. Accession numbers of strain from Pakistan are highlighted in grey. Amino acid position is marked by numbers at the top.

```
                                        10         20         30         40         50         60

PV492552 LSDV_1_NS_2022_PAK       MDRALSIFPG DDDETNERNI NHREKTSGEY GHYEDKLLDL SEEEPNMIKI KNDIKKIINE
OR393178 LSDV/2022/Nohar          MDRALSIFPG DDDETNERNI NHREKTSGEY GHYEDKLLDL SEEEPNMIKI KNDIKKIINE
OR134837 LSDV/Albania/790/2017    MDRALSIFPG DDDETNERNI NHREKTSGEY GHYEDKLLDL SEEEPNMIKI KNDIKKIINE
PQ510117 LSDV/Gaur/Karntaka/2023 MDRALSIFPG DDDETNERNI NHREKTSGEH GHYEDKLLDL SEEEPNMIKI KNDIKKIINE
OR567413 LSDV/Jiling/2022        MDRALSIFPG DDDETNERNI NHREKTSGEH GHYEDKLLDL SEEEPNMIKI KNDIKKIINE
                                 ********** ********** *********: ********** ********** **********


                                        70         80         90        100        110

PV492552 LSDV_1_NS_2022_PAK       RYSNYISIDD DEISDILKDS FISNEEMQIK DFVLRLLVLE KLFQTSVKEC NSLKNIIKRL
OR393178 LSDV/2022/Nohar          RYSNYISIDD DEISDILKDS FISNEEMQIK DFVLRLLVLE KLFQTSVKEC NSLKNIIKRL
OR134837 LSDV/Albania/790/2017    RYSNYISIDD DEISDILKDS FISNEEMQIK DFVLRLLVLE KLFQTSVKEC NSLKNIIKRL
PQ510117 LSDV/Gaur/Karntaka/2023 RYSNYISIDD DEISDILKDS FISNEEMQIK DFVLRLLVLE KLFQTSVKEC NSLKNIIKRL
OR567413 LSDV/Jiling/2022        RYSNYISIDD DEISDILKDS FISNEEMQIK DFVLRLLVLE KLFQTSVKEC NSLKNIIKRL
                                 ********** ********** ********** ********** ********** **********


                                        130        140

PV492552 LSDV_1_NS_2022_PAK       ENHIETIRKN MIVLTKKVDF QTGRSTTL
OR393178 LSDV/2022/Nohar          ENHIETIRKN MIVLTKKVDF QTGRSTTL
OR134837 LSDV/Albania/790/2017    ENHIETIRKN MIVLTKKVDF QTGRSTTL
PQ510117 LSDV/Gaur/Karntaka/2023 ENHIETIRKN MIVLTKKVDF QTGRSTTL
OR567413 LSDV/Jiling/2022        ENHIETIRKN MIVLTKKVDF QTGRSTTL
```

Supplementary Figure S3B; Amino acid sequence alignment of Fusion protein gene with its close relatives in phylogeny. The amino acid changes are highlighted in yellow color. Accession numbers of strain from Pakistan are highlighted in grey. Amino acid position is marked by numbers at the top.

```
                              10             20         30            40         50         60

PV492551 LSDV_1_NS_2022_PAK  STTKKLLQSS IGELFIETGQ TILTDIIDKG PNPFISNMHL KRSVFCSELP DFSCSGSKKI
MT643825 210LSD-249/BUL/16    STTKKLLQSS IGELFIETGQ TILTDIIDKG PNPFISNMHL KRSVFCSELP DFSCSGSKKI
MW656253 LSDV/280-KZN/RSA/2018 STTKKLLQSS IGELFIETGQ TILTDIIDKG PNPFISNMHL KRSVFCSELP DFSCSGSKKI
OR567413 LSDV/Jiling/2022     STTKKLLQSS IGELFIETGQ TILTDIIDKG PNPFISNMHL KRSVFCSELP DFSCSGSKKI
OR393171 LSDV/2022/Surat/N7    STTKKLLQSS IGELFIETGQ TILTDIIDKG PNPFISNMHL KRSVFCSELP DFSCSGSKKI
                              ********** ********** ********** ********** ********** **********

                              70           80          90         100         110        120

PV492551 LSDV_1_NS_2022_PAK  KADNIKKLTE PCIVGRPCFS NRIHNKNHAS IIIDTNYKPV FDKVDNALMR RIALVKFRTH
MT643825 210LSD-249/BUL/16    KADNIKKLTE PCIVGRPCFS NRIHNKNHAS IIIDTNYKPV FDKVDNALMR RIALVKFRTH
MW656253 LSDV/280-KZN/RSA/2018 KADNIKKLTE PCIVGRPCFS NRIHNKNHAS IIIDTNYKPV FDKVDNALMR RIALVKFRTH
OR567413 LSDV/Jiling/2022    KADNIKKLTE PCIVGRPCFS NRIHNKNHAS IIIDTNYKPV FDKVDNALMR RIALVKFRTH
OR393171 LSDV/2022/Surat/N7    KADNIKKLTE PCIVGRPCFS NRIHNKNHAS IIIDTNYKPV FDKVDNALMR RIALVKFRTH
                              ********** ********** ********** ********** ********** **********

                                 130        140        150       160        170         180

PV492551 LSDV_1_NS_2022_PAK  FSQYSNSDSV KNNAAYDDVK PLDENLDMKI QKNYFRYAFLN LLVKWYQKY HIPTMRLFPT
MT643825 210LSD-249/BUL/16    FSQYSNSDSV KNNAAYDDVK PLDENLDMKI QKNYFRYAFLN LLVKWYQKY HIPTMRLFPT
MW656253 LSDV/280-KZN/RSA/2018 FSQYSNSDSV KNNAAYDDVK PLDENLDMKI QKNYFRYAFLN LLVKWYQKY HIPTMRLFPT
OR567413 LSDV/Jiling/2022     FSQYSNSDSV KNNAAYDDVK PLDENLDMKI QKNYFRYAFLN LLVKWYQKY HIPTMRLFPT
OR393171 LSDV/2022/Surat/N7    FSQYSNSDSV KNNAAYDDVK PLDENLDMKI QKNYFRYAFLN LLVKWYQKY HIPTMRLFPT
                              ********** ********** ********** *********** ********* **********

PV492551 LSDV_1_NS_2022_PAK PEAIPDFVFQL
MT643825 210LSD-249/BUL/16    PEAIPDFVFQL
MW656253 LSDV/280-KZN/RSA/2018PEAIPDFVFQL
OR567413 LSDV/Jiling/2022     PEAIPDFVFQL
OR393171 LSDV/2022/Surat/N7   PEAIPDFVFQL
```

Supplementary Figure S3C; Amino acid sequence alignment of NTPase gene with its close relatives in phylogeny. The amino acid changes are highlighted in yellow color. Accession numbers of strain from Pakistan are highlighted in grey. Amino acid position is marked by numbers at the top.

```
OR194148 LSDV/Kurgan/2018      MEGSDNTNTH CWICKDEYNV STNFCNCKNE FKIVHKNCLE EWINFSHDTK CKICNGKYNI
OR393171 LSDV/2022/Surat/N7    MEGSDNTNTH CWICKDEYNV STNFCNCKNE FKIVHKNCLE EWINFSHDTK CKICNGKYNI
MW656253 LSDV/280-KZN/RSA/2018 MEGSDNTNTH CWICKDEYNV STNFCNCKNE FKIVHKNCLE EWINFSHDTK CKICNGKYNI
PV492554 LSDV_1_NS_2022_PAK    MEGSDNTNTH CWICKDEYNV STNFCNCKNE FKIVHKNCLE EWINFSHDTK CKICNGKYNI
                              ********** ********** ********** ********** ********** **********
                                  70         80         90        100        110       120
OR194148 LSDV/Kurgan/2018      KKNKKSCLRW KCSFMYCNIP AICVSLICLL LLPLTILLVK FNLKSMLENI ENRDLITLIS
OR393171 LSDV/2022/Surat/N7    KKNKKSCLRW KCSFMYCNIP AICVSLICLL LLPLTILLVK FNLKSMLENI ENRDLITLIS
MW656253 LSDV/280-KZN/RSA/2018 KKNKKSCLRW KCSFMYCNIP AICVSLICLL LLPLTILLVK FNLKSMLENI ENRDLITLIS
PV492554 LSDV_1_NS_2022_PAK    KKNKKSCLRW KCSFMYCNIP AICVSLICLL LLPLTILLVK FNFKSMLENI ENRDLITLIS
                              ********** ********** ********** ********** **.******* **********
                                   130        140        150       160
OR194148 LSDV/Kurgan/2018      AIAYSLPCVV GFITVIHILI ALYDYYLAAK SDNITYQVYE YI
OR393171 LSDV/2022/Surat/N7    AIAYSLPCVV GFITVIHILI ALYDYYLAAK SDNITYQVYE YI
MW656253 LSDV/280-KZN/RSA/2018 AIAYSLPCVV GFITVIHILI ALYDYYLAAK SDNITYQVYE YI
PV492554 LSDV_1_NS_2022_PAK    AIAYSLPCVV GFITVIHILI ALYDYYLAAK SDNITYQVYE YI
                              ********** ********** ********** ********** **
```

Supplementary Figure S3D; Amino acid sequence alignment of LAP/PHD finger-like protein gene with its close relatives in phylogeny. The amino acid changes are highlighted in yellow color. Accession numbers of strain from Pakistan are highlighted in grey. Amino acid position is marked by numbers at the top.

```
                                       10               20           30               40           50          60
PV492553 LSDV_1_NS_2022_PAK  MGSSFTVPEK IKISNRPKKE TMEMRLNVDN MYEQIKTLNQ INDELYIGII NEEKKKQLIK
OQ588787 LSDV/02/KASH/IND/2022 MGSSFTVPEK IKISNRPKKE TMEMRLNVDN MYEQIKTLNQ INDELYIGII NEEKKKQLIK
KY702007 SERBIA/Bujanovac/2016 MGSSFTVPEK IKISNRPKKE TMEMRLNVDN MYEQIKTLNQ INDELYIGII NEEKKKQLIK
OM033705 LSDV/Thailand/YST/2021MGSSFTVPEK IKISNRPKKE TMEMRLNVDN MYEQIKTLNQ INDELYIGII NEEKKKQLIK
OR567413 LSDV/Jiling/2022       MGSSFTVPEK IKISNRPKKE TMEMRLNVDN MYEQIKTLNQ INDELYIGII NEEKKKQLIK
                               ********** ********** ********** ********** ********** **********
                                   70           80          90          100            110      120
PV492553 LSDV_1_NS_2022_PAK  QFPEFLFIES GPGNLHKVIR SKYNNDKKYC CKSMNLFSYW QDNNGNITDF YKPNSILSSC
OQ588787 LSDV/02/KASH/IND/2022 QFPEFLFIES GPGNLHKVIR SKYNNDKKYC CKSMNLFSYW QDNNGNITDF YKPNSILSSC
KY702007 SERBIA/Bujanovac/2016 QFPEFLFIES GPGNLHKVIR SKYNNDKKYC CKSMNLFSYW QDNNGNITDF YKPNSILSSC
OM033705 LSDV/Thailand/YST/2021QFPEFLFIES GPGNLHKVIR SKYNNDKKYC CKSMNLFSYW QDNNGNITDF YKPNSILSSC
OR567413 LSDV/Jiling/2022       QFPEFLFIES GPGNLHKVIR SKYNNDKKYC CKSMNLFSYW QDNNGNITDF YKPNSILSSC
                               ********** ********** ********** ********** ********** **********
                                    130          140           150         160           170         180
PV492553 LSDV_1_NS_2022_PAK  DPDIQNSGFC DDTLLSWCDT ATQSNSKLCY DWINSALNRN NSSSEKLING LITTCSNNAN
OQ588787 LSDV/02/KASH/IND/2022 DPDIQNSGFC DDTLLSWCDT ATQSNSKLCY DWINSALNRN NSSSEKLING LITTCSNNAN
KY702007 SERBIA/Bujanovac/2016 DPDIQNSGFC DDTLLSWCDT ATQSNSKLCY DWINSALNRN NSSSEKLING LITTCSNNAN
OM033705 LSDV/Thailand/YST/2021DPDIQNSGFC DDTLLSWCDT ATQSNSKLCY DWINSALNRN NSSSEKLING LITTCSNNAN
OR567413 LSDV/Jiling/2022       DPDIQNSGFC DDTLLSWCDT ATQSNSKLCY DWINSALNRN NSSSEKLING LITTCSNNAN
                               ********** ********** ********** ********** ********** **********
                                    190              200         210           220         230           240
PV492553 LSDV_1_NS_2022_PAK  TPICNIFLHC LRVKNTETFD NVIDYILYSQ SDDFKRTYMK CSYPSNKIIK ESLKFFEARE
OQ588787 LSDV/02/KASH/IND/2022 TPICNIFLHC LRVKNTETFD NVIDYILYSQ SDDFKRTYMK CSYPSNKIIK ESLKFFEARE
KY702007 SERBIA/Bujanovac/2016 TPICNIFLHC LRVKNTETFD NVIDYILYSQ SDDFKRTYMK CSYPSNKIIK ESLKFFEARE
OM033705 LSDV/Thailand/YST/2021TPICNIFLHC LRVKNTETFD NVIDYILYSQ SDDFKRTYMK CSYPSNKIIK ESLKFFEARE
OR567413 LSDV/Jiling/2022       TPICNIFLHC LRVKNTETFD NVIDYILYSQ SDDFKRTYMK CSYPSNKIIK ESLKFFEARE
                               ********** ********** ********** ********** ********** **********
```

Supplementary Figure S3E; Amino acid sequence alignment of VLTF-1 gene with its close relatives in phylogeny. The amino acid changes are highlighted in yellow color. Accession numbers of strain from Pakistan are highlighted in grey. Amino acid position is marked by numbers at the top.

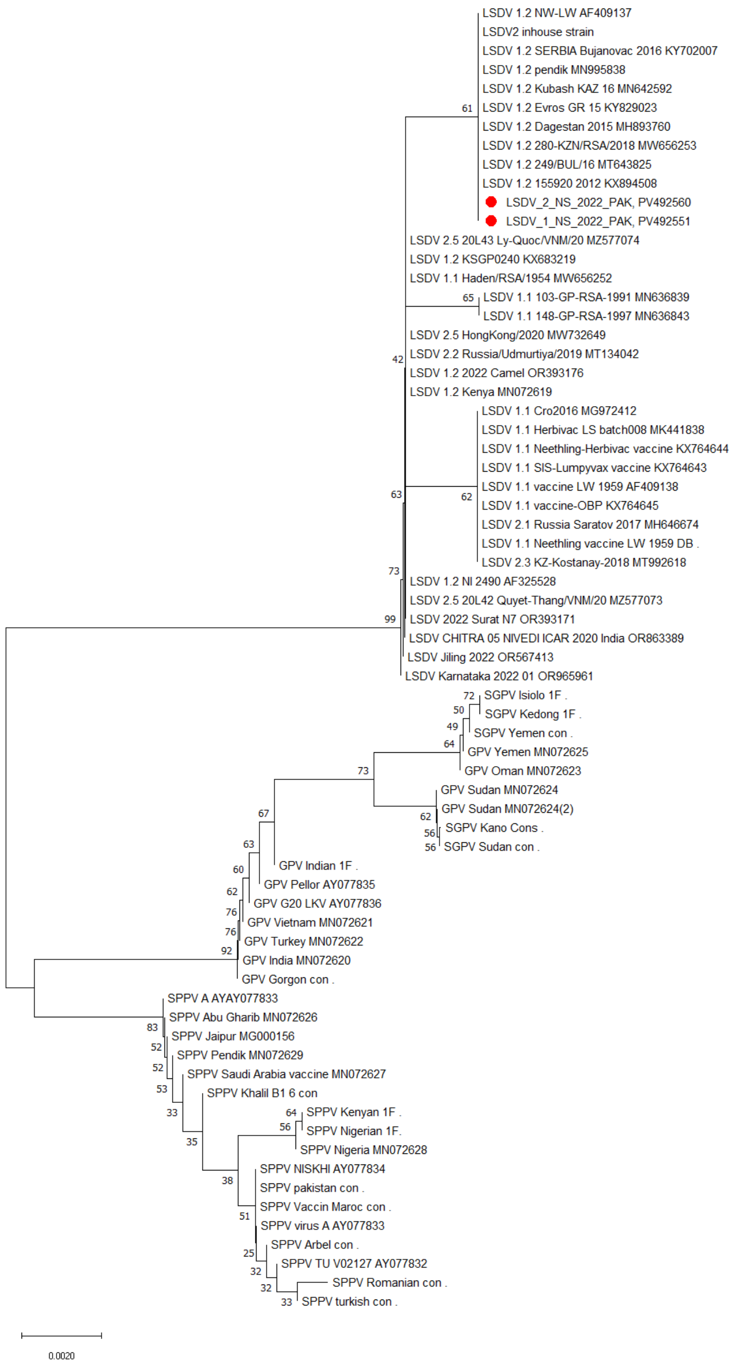

Figure S4A; Phylogenetic tree representing *NTPase* gene of isolates from Pakistan *LSDV_1_NS_2022_PAK*, PV492551 and *LSDV_2_NS_2022_PAK*, PV492560, indicating their close relation to Russian and Bulgarian strains of LSDV. The tree was generated by Neighbor joining method.

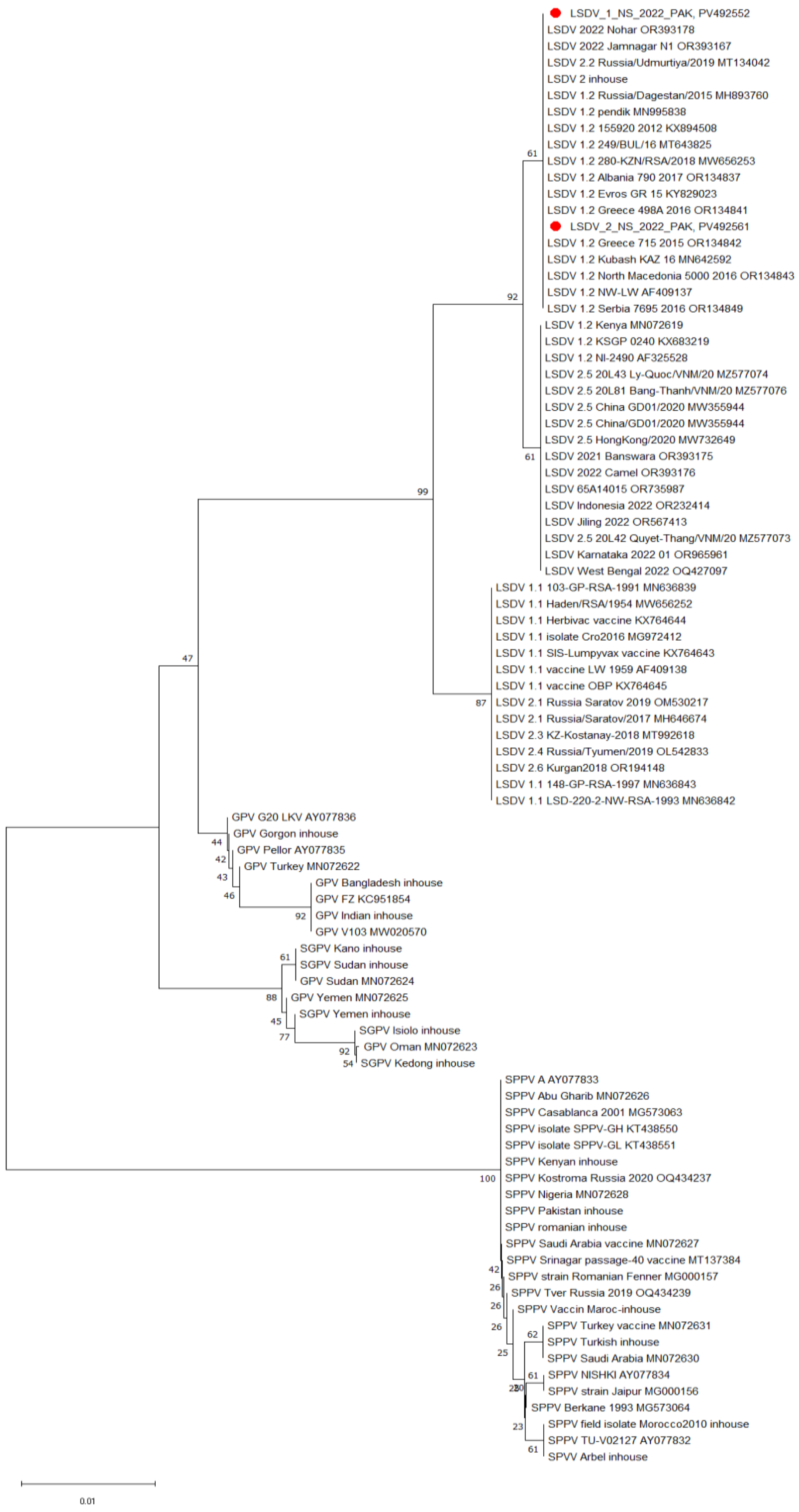

Figure S4B; Phylogenetic tree representing *RPO132* gene from isolates of Pakistan *LSDV_1_NS_2022_PAK*, PV492552 and *LSDV_2_NS_2022_PAK*, PV492561 indicating their close relation to Russian, Balkan, and Indian strains of LSDV. The tree was generated by neighbor joining method; however, the phylogenetic placement of both isolates remained the same by maximum likelihood method.

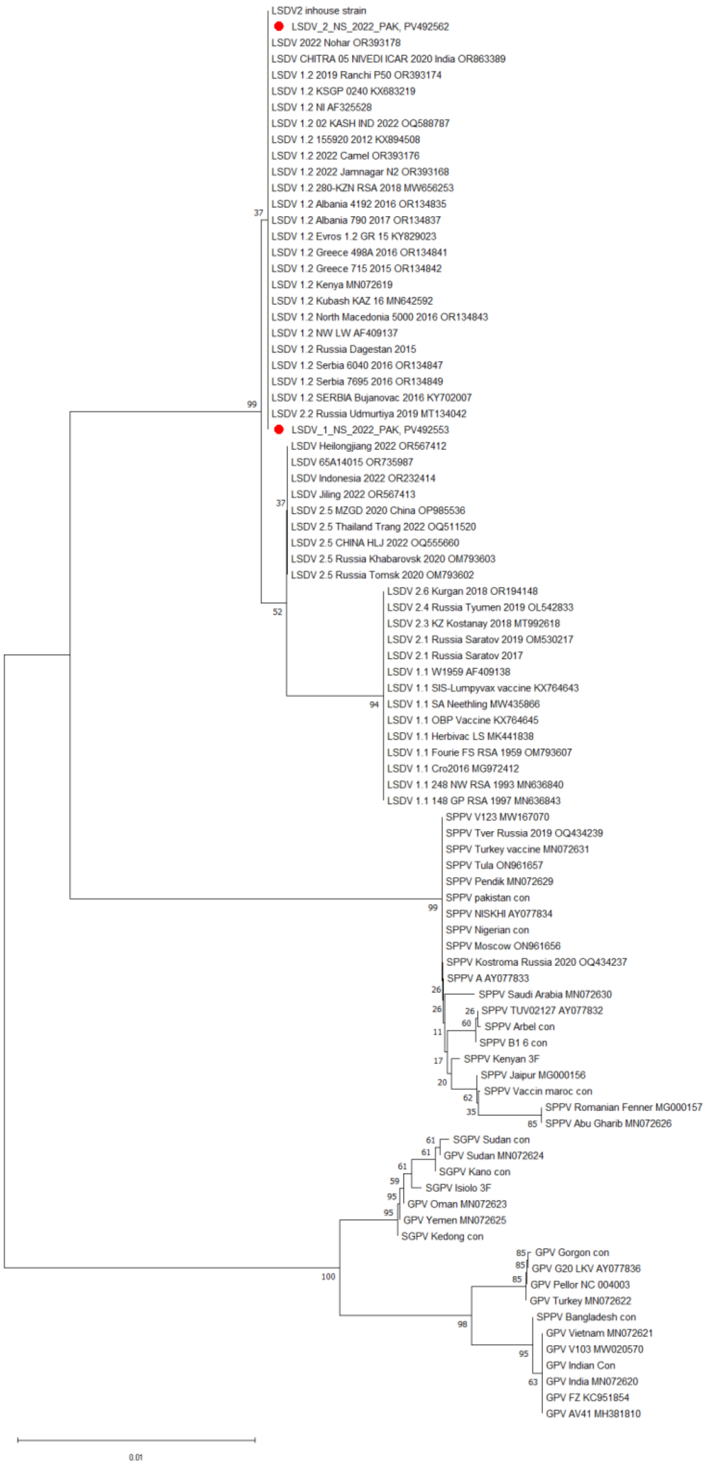

Figure S4C; Phylogenetic tree representing *VLTF-1* gene from isolates of Pakistan *LSDV_1_NS_2022_PAK*, PV492553, *LSDV_2_NS_2022_PAK*, PV492562, indicating their close relation to Russian, Balkan, and Indian strains of LSDV. The tree was generated by neighbor joining method; however, the phylogenetic placement of both isolates remained the same by maximum likelihood method.

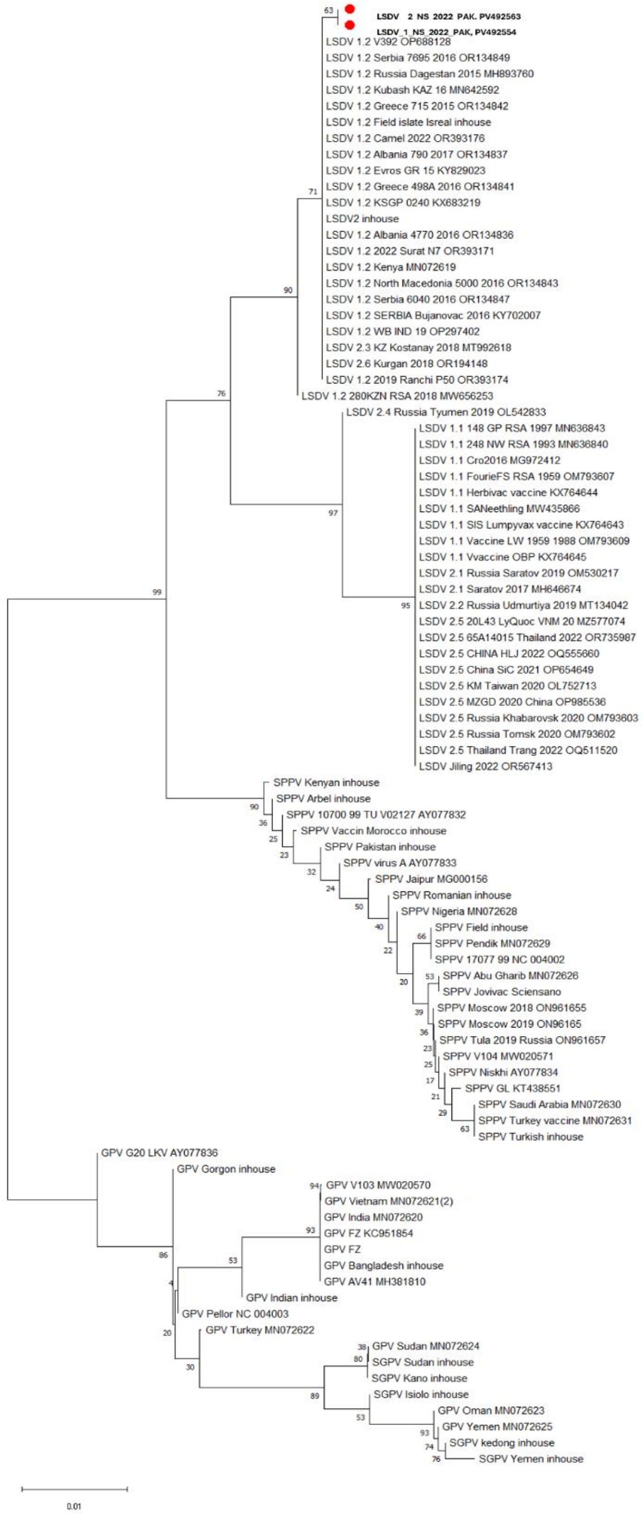

Figure S4D; Phylogenetic tree of *LAP/PHD finger like protein* gene of isolates from Pakistan LSDV_1_NS_2022_PAK, PV492554, LSDV_2_NS_2022_PAK, PV492563, indicating their close relation to Indian and Balkan strains of LSDV. The tree was generated by neighbor joining method; however, the phylogenetic placement of both isolates remained the same by maximum likelihood method.

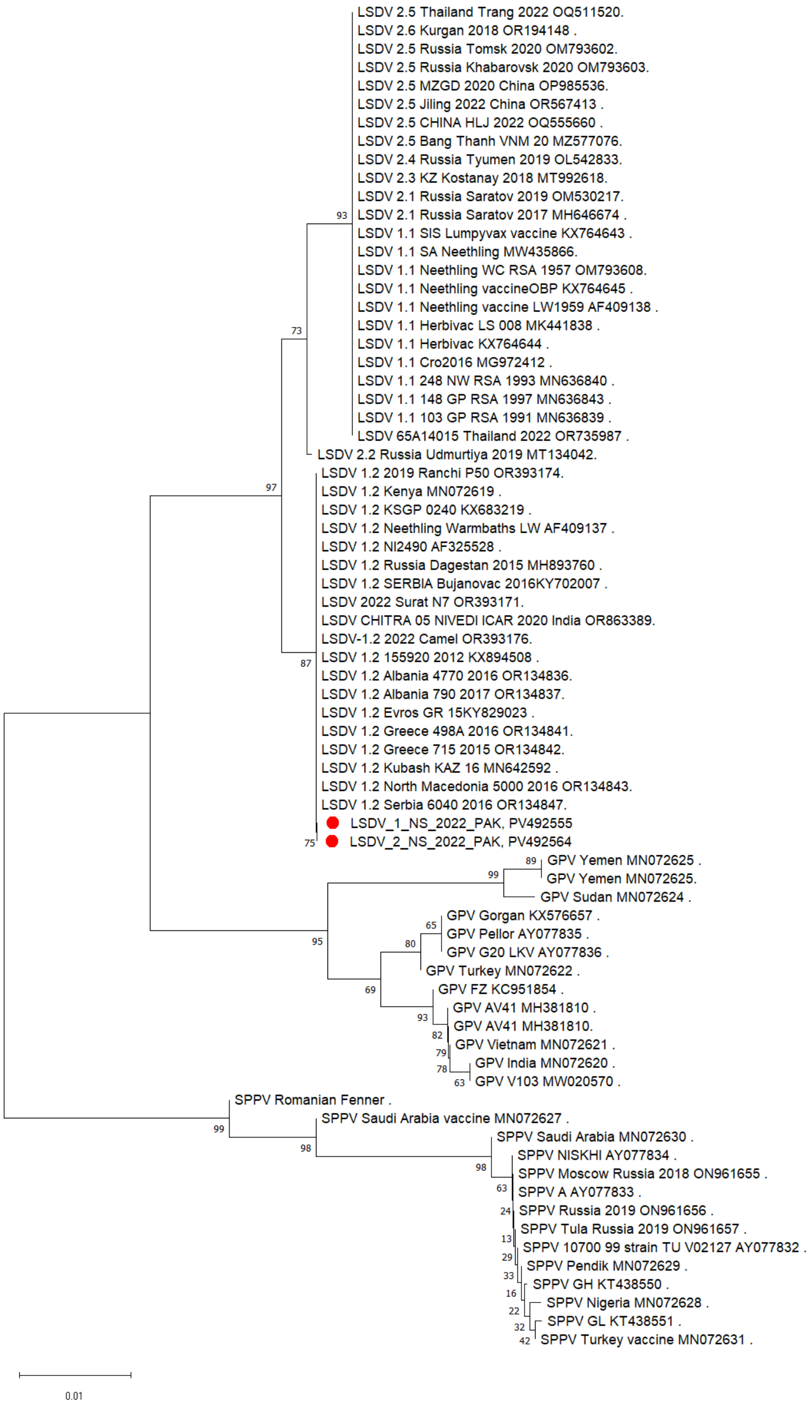

Figure S4E; Phylogenetic tree representing *Ser/Thr Kinase* gene from isolates of Pakistan LSDV_1_NS_2022_PAK, PV492555, LSDV_2_NS_2022_PAK, PV492564 , indicating their close relation to Russian and Albanian strains of LSDV. The tree was generated by neighbor joining method; however, the phylogenetic placement of both isolates remained the same by maximum likelihood method.
